# Developing a dataset to track aid for reproductive, maternal, newborn and child health, 2003–2013

**DOI:** 10.1038/sdata.2017.38

**Published:** 2017-03-28

**Authors:** Christopher Grollman, Leonardo Arregoces, Melisa Martinez-Alvarez, Catherine Pitt, Timothy Powell-Jackson, Justine Hsu, Giulia Greco, Josephine Borghi

**Affiliations:** 1 Department of Global Health and Development, London School of Hygiene and Tropical Medicine, 15-17 Tavistock Place, London WC1H 9SH, UK; 2 Department of Health Systems Governance and Financing, World Health Organization, 20 avenue Appia, 1211 Geneva 27, Switzerland

**Keywords:** Health care economics, Health policy

## Abstract

We created a dataset to generate estimates of donor-reported ‘official development assistance’ and private grants (ODA+) to reproductive, maternal, newborn and child health (RMNCH) by donor, recipient country and activity type over the period 2003–2013. We collected disbursement information from the Organisation for Economic Co-operation and Development Creditor Reporting System (CRS) in January 2015. All 2.1 million records across all sectors were coded based on donor name, project title, short and long descriptions, and CRS code describing the purpose of the disbursement. We classified records according to the degree to which they would promote attainment of Millennium Development Goals 4 and 5 (reproductive and sexual health, maternal and newborn health, and child health). We also classified records according to whether they supported prenatal and neonatal health (PNH). The dataset includes project funding as well as allocating shares of general budget support, health sector support and basket funding. The data can be used to analyse resource flows to RMNCH or to other purposes or beneficiaries of ODA+.

## Background & Summary

The Countdown to 2015 was established to monitor countries’ progress towards Millennium Development Goals (MDGs) 4 and 5 in relation to mortality, service coverage, equity and financing, with particular focus on the 75 countries where more than 95% of all maternal and child deaths occur. To accelerate progress towards the MDGs, donors made pledges to increase funding to 75 low- and middle-income countries considered by Countdown to have the greatest burden of maternal and child ill-health (http://www.who.int/pmnch/activities/secretariats/countdown/en/). Countdown tracks disbursements reported by donors to the Creditor Reporting System (CRS) maintained by the Organisation for Economic Co-operation and Development (OECD). The initiative began by tracking ‘official development assistance’ (ODA) disbursements to maternal, newborn and child health (MNCH) in 2003 and assessed whether funding levels were associated with burden of disease, to help hold donors to account for their pledges and commitments^1–7^. In 2009, the tracking exercise expanded to include reproductive and sexual health^3^ and disbursements of private grants from the Bill and Melinda Gates Foundation, which together with ODA we refer to as ODA+.

Countdown is not the only initiative to track resource flows for reproductive, maternal, newborn and child health (RMNCH). Since 2002 the Resource Flows project of the United Nations Population Fund (UNFPA) and the Netherlands Interdisciplinary Demographic Institute (NIDI, http://www.resourceflows.org/) have been tracking flows to ‘population assistance’, which has some overlap with reproductive, maternal and newborn health. In 2010 the Institute for Health Metrics and Evaluation (IHME) began tracking flows of ‘development assistance for health’, split into ‘focus areas’ including ‘maternal health’ and ‘newborn and child health’^8^. The Partnership for Maternal, Newborn and Child Health (PMNCH) began tracking financial commitments (not disbursements) for RMNCH in 2011, focused on the 49 recipient countries of the Every Woman Every Child Global Strategy and since expanding to cover the 75 Countdown priority recipient countries^9^. Our approach can be distinguished from these others by our RMNCH coding method and framework, which enable the breakdown of resources by funding type (i.e. general budget support, health sector budget support, basket or pooled funding, or projects), activity type for projects (e.g. nutrition, immunisation) and by beneficiary group, and our assessment of whether funds are targeted to need^4,6,7,10^. We have also compared in detail the Countdown, IHME and PMNCH approaches and how they build on the CRS (forthcoming). In addition, we also developed a separate framework to categorize records mentioning prenatal and neonatal health (PNH) in greater depth.

We used data on ODA+ disbursements from all donors to all recipient countries in the CRS. Each record in the CRS contains information on the donor, recipient country, disbursement amount, channel and flow of the disbursement, and information regarding its purpose. To estimate the total value of funding supporting RMNCH both through dedicated projects and through investments in health systems, we manually coded records based on descriptive information provided by the donors, using a pre-defined RMNCH coding framework^1,3,7^. To examine funding supporting PNH in greater depth, we developed and applied an automated key term search approach to identify all records mentioning PNH, and then manually coded this sub-set of records using a pre-defined PNH coding framework^6,11^. We looked at records from all aid sectors to ensure we identified health funding reported with non-health purpose codes within the CRS^3,4,7^.

Countdown has used estimates of ODA+ disbursements to report on trends in ODA+ to MNCH^2,4,5,7^, to reproductive and sexual health^3^, to prenatal and neonatal health^11^, and to RMNCH^1,10^. Analyses are underway to study alignment and harmonisation of ODA+ to RMNCH, determinants of fragmentation, ODA+ to Latin America and the Caribbean and ODA+ for family planning. This paper presents the final Countdown ODA+ Dataset (Data Citation 1), and explains how it was generated. The present dataset can be used to replicate and build on previous analyses, to analyse aid flows to RMNCH by donor or by recipient, by lending type and by channel of funding. The dataset can also be used to compare RMNCH funding to that of other sectors or health conditions, for comparison with domestic expenditure or to investigate financing gaps.

## Methods

**Figure 1:**
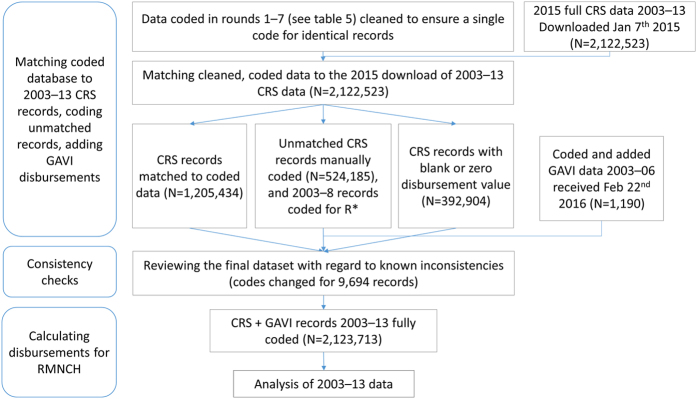
Process of creating a fully coded dataset of ODA+ for RMNCH.

We begin by describing the data sources used to generate the dataset, and then outline the approach used to code for RMNCH, steps taken to update the coding, and lastly steps taken to allocate disbursements for RMNCH and to adjust for inflation. The process is illustrated in Fig. 1.

### Data sources

As detailed below, we obtained data on ODA+ disbursements from the OECD CRS and supplemented this with data obtained directly from the Vaccine Alliance (GAVI) on its disbursements made in 2003–06. Our analysis of the value of ODA+ supporting RMNCH was also informed by additional data on demographics, health conditions, and health financing.

### Data on ODA+ disbursements in the Creditor Reporting System

The OECD CRS is a database to which donors of official development assistance (ODA grants, grant-like and loans), other official flows and private grants report their commitment and disbursement activities, and is described at http://www.oecd.org/dac/stats/methodology.htm. Donors include members of the OECD Development Assistance Committee (DAC), non-DAC bilateral donors, multilateral development agencies (including development banks, the International Monetary Fund, and specialised agencies of the United Nations), global health initiatives (the Vaccine Alliance, GAVI, and the Global Fund to Fight AIDS, Tuberculosis and Malaria, GFATM) and a private foundation (Bill and Melinda Gates Foundation). Recipients are defined by the CRS as all ‘developing countries’ eligible to receive ODA. These include all ‘least developed countries’ as defined by the United Nations and all low- and middle-income countries defined by the World Bank, except any that are members of the G8 or members or agreed future members of the European Union; full details are given at http://www.oecd.org/dac/stats/daclist.htm. Supplementary File 1 shows the full list of donors and the years for which they appear in the dataset. The full list of recipient countries is shown in Supplementary File 2, which also highlights the 75 countries that Countdown considered to be of greatest priority because of their burden of maternal and child ill-health. The CRS data can be downloaded from the OECD website at https://stats.oecd.org/Index.aspx?DataSetCode=CRS1 (accessed on 7th January 2015). CRS data are copyright OECD and free to use for any purpose with acknowledgement of their source. Data on ODA, private grants and other official flows are uploaded to the CRS website by the OECD twice each year, covering disbursements made up until two calendar years prior. Donors are able to add, remove and edit the data reported to the CRS, and these changes are reflected in subsequent uploads by OECD to the website.

In each round data were downloaded from the CRS website as comma-separated value files (using the ‘Related files’ option in the ‘Export’ menu on the CRS website). Up until the last round, data were converted into Excel spreadsheets for coding and analysis. For the last round of data coding and to generate the final Countdown ODA+ Dataset, we downloaded the CRS data for the years 2003–2013 and imported these data into Microsoft SQL Server Management Studio (SSMS; Microsoft Corporation, 2014) for data management. The downloaded data covers all ODA and private grant disbursements (collectively termed ODA+) and excludes equity investments and other official flows. The Countdown ODA+ Dataset (Data Citation 1) is based on the download of the CRS on January 7th 2015 covering the period 2003–2013 (hereafter referred to as the ‘2015 full CRS’).

Values of disbursements in the 2015 full CRS were in current US dollars for the respective years of disbursement. Deflators to convert these to constant 2013 US dollars, taking into account inflation in the original currency, were obtained from the OECD website at http://www.oecd.org/dac/stats/informationnoteonthedacdeflators.htm; the ‘Total DAC’ deflator was used where a national estimate was not available.

The Countdown ODA+ Dataset (Data Citation 1) contains 92 fields, which describe the nature of each of the disbursements. These comprise all the fields reported in the CRS; and additional fields generated through our data processing and coding. These fields are listed in the data dictionary available at the data deposit (see Data Records below).

### Data on ODA disbursements by GAVI in 2003–06

We obtained data on disbursements from GAVI for the years 2003–06 directly from GAVI (D. Mocova, GAVI, personal communication, 22 February 2016); data for later years were available from the CRS. The data from GAVI comprised the disbursement amount, recipient name and a project title; where the recipient was ‘GAVI eligible countries’ we divided the disbursement between all GAVI recipients receiving disbursements in that year in proportion to their share of disbursements to all named recipients. We added the resulting 1,190 records to the 2015 full CRS dataset.

### Data to inform estimates of ODA+ for RMNCH

We also obtained data from a variety of sources on demographics, health conditions, and health financing (Supplementary File 3). We used this data to inform our estimates of the value of ODA+ supporting RMNCH in those RMNCH codes calculated using the recipient country-specific values in Table 1. This is described in more detail below in the section ‘Proportions of disbursements assigned to R*, MNH, CH’.

### Coding records for RMNCH and for PNH

#### RMNCH coding framework

A set of codes was initially developed for maternal, newborn and child health-related expenditures^7^ and subsequently extended in 2009 to capture reproductive and sexual health expenditures^3^. Maternal and newborn health expenditures include activities to restore, improve, and maintain the health of women and their newborns during pregnancy, childbirth and the first month of life^7^. Expenditures for child health include activities to restore, improve and maintain the health of children up to five years of age^7^. Where age was not specified, we assume the term ‘child’ referred to children aged under five years. Reproductive health and sexual health expenditures (termed R*) include expenditures on family planning, sexual health and sexually transmitted infections, including HIV^3^. In addition to funding exclusively earmarked for RMNCH, we also identified other activities thought to benefit RMNCH, including funds for general health systems or health care, general budget support and basket or sector funding and some condition-specific funding (for example, funds for malaria and HIV programmes).

Table 2 (available online only) shows the complete list of RMNCH code names and definitions. The coding scheme has been applied over time with minor alterations to definitions in order to increase clarity, assist in classifying borderline cases and make the scheme as exhaustive as possible. Each disbursement record was assigned a single code: multiple codes cannot be assigned to a single record.

#### Assigning RMNCH codes

RMNCH codes were assigned manually through a review of information reported by donors to the CRS. Across the duration of the Countdown initiative, the most important fields were the following five fields: Donor name, Project title, Short description, Long description and Purpose code (Table 3).

Whilst in prior rounds each record was coded individually, in the last round of coding each unique combination of these five fields was coded, instead of coding individual records. This increased the consistency and efficiency of coding.

In 2013, in order to reduce the cognitive load and increase the speed of manual review, flags were added to the data to be coded to indicate the presence of key terms related to the RMNCH codes to be assigned. These flags were generated through searches for terms in the three descriptive fields: Project title, Short description and Long description. For example, the flag for terms related to Integrated Management of Childhood Illnesses (RMNCH code 414) occurred for any project where any of the three fields contained the terms ‘IMCI’, ‘integrated child’, ‘ICCM’, ‘IMNCI’, ‘PCIME’ or ‘EPI ’.

Codes were then assigned according to the following standard approach to reviewing the five fields:

- Assume that the purpose code is correct unless two of three of the remaining fields indicate otherwise. Where the descriptive fields were blank, we relied on the purpose code. Table 4 shows the relationship between purpose codes in the health and population sectors (sector codes 120 and 130 in the CRS) and RMNCH codes, in the absence of any information in the other descriptive fields.
- Assume that the long description is correct if it provides further specification but does not contradict the other three fields (e.g. specifies prevention of mother-to-child transmission on a project otherwise described as HIV/AIDS).
- For records in sector codes other than 120 and 130, assume that the project is not related to RMNCH unless either:
- At least one of the three fields indicate that the project is relevant to RMNCH and the two other fields do not contradict this, or
- Two of the three fields indicate that the project is relevant to RMNCH.

Where multiple RMNCH codes were possible, the code assigned was generally that which most accurately fit the project description including the purpose code. In some cases where more than one specific code was possible we assigned a code that was less specific but reflected the beneficiaries of the various possible options. For instance, a project described as for ‘Immunisation, nutrition and Water, Sanitation & Hygiene (WASH)’ should receive code 431—maternal and child health—as both immunisation and nutrition activities benefit these groups. A precise fit was not always possible.

Outside the health and population sectors (CRS sector codes 120/130) the purpose codes most frequently coded as relevant to RMNCH (i.e. not 0) were 51010 (budget support) and 15250 (action against landmines).

#### RMNCH allocation factors

Where a record was fully described by one of our RMNCH codes, it was assigned an allocation factor of 1. Sometimes a single record described both activities relevant to RMNCH and therefore merited a non-zero RMNCH code and also activities that were not relevant to RMNCH and therefore merited a zero code. Such records received the appropriate non-zero RMNCH code and an allocation factor of less than 1, reflecting the proportion of the record that was disbursed to the RMNCH-relevant activities. The allocation factor was calculated as the number of activities described that would receive a non-0 RMNCH code as a share of all activities described. For example, a record providing funds for ‘Five basic services: education, health, community development, agriculture and roads’ would be coded as 440 (general health system—primary health care) with an allocation factor of 0.2, since health was one of five activities listed.

#### Coding records for PNH

In 2012 we introduced an additional analysis, using the same underlying CRS data to identify and further categorize records mentioning prenatal and neonatal health (PNH) or directly relevant activities, as described elsewhere^11^. This analysis took a different approach to the RMNCH analysis: rather than reading all records for relevance to PNH, we conducted a search of the three descriptive text fields to identify records containing a key term related to PNH, then reviewed all records with at least one key term. We aimed to identify records that mentioned the health of the newborn or fetus, or which indicated that they supported interventions in pregnancy or in the first four weeks of life that are proven to improve or maintain the health of the baby before, during, or in the first 28 days following birth. We developed key terms by reviewing scientific literature; generating a list of general terms, conditions and diseases, and interventions and programmes meeting our criteria; and then carefully refining our terms^6^. We repeated this exercise when preparing the final Countdown ODA+ Dataset, at which point we expanded the list of key terms to increase their sensitivity and used 135 search terms in seven languages (English, French, Dutch, Spanish, Portuguese, Italian, German). We conducted the key-term search in SSMS.

We reviewed and classified the subset of records containing at least one PNH search term in any of the three descriptive fields. Records with a blank or zero disbursement value were coded as ‘zero’. In the last round of coding, a single coder read and individually coded the records with non-zero disbursement values. Records were coded as ‘misclassified’ if they did not in any way support or mention prenatal or neonatal health. The remaining non-zero, correctly classified records mentioning PNH were then coded in two ways. First, records were categorized as supporting either (1) non-research or, (2) research activities. Second, records were classified as either (1) exclusively benefiting PNH, or (2) also benefiting other population groups, such as mothers or children older than one month.

#### Rounds of RMNCH and PNH coding

Several rounds of coding were conducted during the course of the Countdown project (Table 5). At each round, the most up-to-date CRS data were downloaded. Rounds 1–4, 6 and 7 involved coding all new data for the full range of RMNCH codes being used at the time (MNCH for rounds 1–3 and RMNCH for rounds 4, 6 and 7). Round 3 additionally involved updating data for rounds 1 and 2 by adding and coding newly reported data for those years for some donors which had not previously reported any disbursements in those years, and by replacing the previously-reported commitments data from the International Development Association (IDA) with the disbursements data, which it provided for the first time while also dramatically changing the way in which it reported its funding. Round 5 involved coding for PNH only and did not involve assigning RMNCH codes. Finally, round 7 involved assigning RMNCH codes for 2013, coding records from 2003–2013 for PNH, and also reviewing records from 2003–2008 coded during rounds 1–3 to determine whether an R* code should be assigned. This R* coding was limited to those records that had initially received the RMNCH code 0 (excluded as not relevant to MNCH, though potentially still relevant to R*), or 421 (for reproductive, maternal and newborn health including safe motherhood) because this code had the most likely overlap with the reproductive and sexual health codes.

In rounds 1–4 and 6, analysis of RMNCH disbursements was done for the years of data that had been newly coded, reflecting disbursements that were available from the CRS at that time. For analysis of time trends in these rounds, the coded data from previous rounds was used without including any updated disbursements since reported to the CRS, except in round 3 where updated data were used for the International Development Association, Italy, Finland, UNFPA and UNAIDS. Donors frequently update their reports to the CRS, and relying on data coded in earlier rounds for time trends meant that new donors were not included in trend analyses, nor were additions or adjustments to project descriptions/disbursement amounts reflected for donors that had reported in the past. Following the coding in round 7 we checked the consistency of previous rounds of coding, and updated some codes for years 2003–2012. As outlined below, the records in the Countdown ODA+ Dataset for the years 2003–2013 correspond to those reported in the full CRS download of January 7th 2015, regardless of what records were coded in previous coding rounds. In round 7, we conducted the PNH coding for all records in the 2003–2013 Countdown ODA+ Dataset; to inform this final coding we transferred codes from the previous PNH coding where records had the same donor name, purpose code and three descriptive fields as a record previously coded.

Six different sets of coders were involved in the coding rounds (Table 5). Round 1 had two coders double-coding all records for RMNCH; rounds 2–4 had a single coder for RMNCH; round 5 had two coders double-coding all records for PNH; round 6 had four coders for RMNCH coding separate sections, to reduce coding time, with consistency checks conducted (see Technical Validation below), and one coder for PNH; and round 7 had a single coder for RMNCH and two coders for PNH.

### Matching the RMNCH-coded records to 2015 full CRS, and coding unmatched records in the 2015 full CRS

At the end of round 7 we had a set of coded records from all the rounds of coding (rounds 1–7). In order to transfer these codes to the 2015 full CRS, we cleaned the records from round 1–7 to ensure consistency and then matched these data to the 2015 full CRS, and coded any records in the 2015 full CRS that did not match to the previously RMNCH-coded records, as detailed below. This process is illustrated in Supplementary File 4.

#### Cleaning historical RMNCH-coded records

To assure consistency in the coded records from rounds 1–7, we identified records that were identical on five key fields (CRS purpose code, project title, short description, long description and donor), but had been assigned different RMNCH codes between or within coding rounds. There were 105,759 such records (7.4% of all coded records), with 8,770 distinct combinations of the five descriptive fields. If five projects were identical, and four received the same code while the fifth received a discrepant code, all five are included in this 105,759.

To reconcile the differences, we adopted the following approach:

1. If the project had a code assigned in round 7, prefer that code, as the final round of coding was based on the most detailed coding descriptions resulting from discussion with two of the previous coders.
2. Manually review and re-code any remaining identical records with discrepant RMNCH codes.

Following this reconciliation, we had a set of data coded in rounds 1–7 for the years 2003–2013 containing 1,438,307 records, with 636,992 distinct combinations of the five key fields. Each record had an RMNCH code and an allocation factor.

#### Matching RMNCH-coded records to the 2015 full CRS

The 2015 full CRS dataset contained 2,122,523 records for 2003–2013. The disbursement value was zero or null for 392,904 records (19%), which were assigned an RMNCH code of 999. We could not use CRS project identifiers to match projects, because the CRS did not maintain the same project identifiers for a given record from one update to the next and because they were not exclusive between projects. To code the remaining 1,729,619 CRS records we first compared them to the records coded in rounds 1–7, based on the five key fields mentioned above. Second, where a record coded in round 1–7 matched a record in the 2015 full CRS, we applied the RMNCH code to the corresponding record in the 2015 full CRS.

Ideally, all records in the 2015 full CRS would have matched coded records. However, there are a variety of reasons why this was not the case:

- Donors added data for 2003–2012 that had not previously been reported or coded.
- Donors made changes to project description fields, for example, suffixes or prefixes were added to otherwise identical description fields, and there were changes to maximum field lengths over time.
- Diacritic accents had sometimes been parsed differently in different years.

Given these constraints, we made some adjustments to the coded records and the 2015 full CRS to minimise missed matches. These adjustments comprised:

- Harmonising as the string ‘BLANK’ all null fields and fields containing certain descriptions (‘NULL’, ‘UNKNOWN’, ‘#NAME?’, ‘#EMPTY’, or <2 characters long).
- Replacing accented characters with non-accented characters.
- Truncating the project title, short description and long description to 250 characters.
- Replacing as 0 any null values for purpose code.

To avoid missing matches due to extra spaces or punctuation changes, for those records that did not match on the adjusted fields, we further attempted to match on the same fields using only alpha-numeric characters (a–z, 0–9).

The matching process resulted in 1,205,434 records in the 2015 full CRS receiving a code (57%). There remained 524,185 unmatched CRS records (25%) to which we added 1,190 records for GAVI covering disbursements in 2003–2006 (Table 6). We coded these 525,375 records by manual review, using the methods outlined above.

### Reviewing the final dataset with regard to known inconsistencies

We undertook certain investigations to assess the reliability and accuracy of the coding, described in the Technical Validation section below. As a result, we changed the RMNCH code for 9,645 matched records and 49 non-matching records, making 0.5% of the total.

### Calculating disbursements to RMNCH and PNH

Once all records had RMNCH codes and allocation factors assigned, disbursements to child health, maternal and newborn health and reproductive and sexual health were calculated using the disbursement value, the allocation factor and the disbursement rule for the relevant RMNCH code, as detailed below.

#### Proportions of disbursements assigned to R*, MNH, CH

For each RMNCH code, we developed a rule to assign a proportion of the disbursement value to each of three mutually exclusive categories—child health, maternal and newborn health and reproductive and sexual health—based on the extent to which the disbursement supported each category. For example, for records assigned code 415 for child vaccinations excluding polio, 100% of the value was assigned to child health, 0% to maternal and newborn health and 0% to reproductive and sexual health (Table 1). For records assigned code 440 for general health system support to primary health care, 40% of the value was assigned to child health and 8.4% to maternal and newborn health, with 0% to reproductive and sexual health. For records assigned code 434 for HIV/AIDS (generic), 0% was assigned to maternal and newborn health, while the proportions assigned to child health and reproductive and sexual health varied by recipient country, based on the proportion of the HIV-positive population that was, respectively, children aged 0–4 and women aged 15+ (Table 1). These disbursement rules were based on a set of assumptions and data sources identified during the first iterations of the Countdown resource-tracking exercise to focus on MNCH^7^ and R*^3^ respectively.

For general budget support, we obtained country-level proportions of government spending for health out of total government spending from the World Health Organization Global Health Expenditure database (http://apps.who.int/nha/database, accessed on 4 April 2016). We then multiplied that proportion by the proportion of health system funds estimated to benefit child health and MNH respectively. The proportion of health systems funds and basket or sector funding assumed to benefit child health and MNH was fixed across countries and based on the literature^7^. The proportion of funding for non-specified infectious diseases and other health conditions that we assumed to benefit child health was based on the year-country-specific proportion of under-5-year-olds in the national population^12^. We used region-specific malaria incidence rates and data on insecticide-treated net use by children under 5 to estimate how much anti-malaria spending benefits child health. The proportion of unspecified anti-malaria funding assumed to benefit MNH was fixed^7^. To allocate generic anti-HIV funding to child health, we used estimates of the share of the total population living with HIV infection in that country in that year that was aged 0–14 (ref. 13) and the proportion of 0–14 year-olds who were aged 0–4 (ref. 12). Country-level, year-specific estimates of the proportion of people living with HIV who were women aged 15+ (ref.^13^) were applied to disbursements for generic anti-HIV funding to estimate the benefit to R*. For disbursements related to sexually transmitted infections, we estimated the proportion that supported R* using region-level estimates of the proportion of people living with any of four sexually transmitted infections who were women^14^ combined with estimates of the proportion of treatment costs that pertained to treating women^15^.

Record-specific disbursement values for CH, MNH and R* were calculated by multiplying:

Disbursementvalue×DisbursementruleforRMNCHcode×RMNCHallocationfactor

#### Value of records supporting PNH

In the analyses conducted, the whole disbursement value of records mentioning PNH key terms and coded as correctly classified was assessed^6,11^. No calculations were performed and no additional fields beyond those in the 2015 full CRS are included in the dataset.

#### Assigning regional and unspecified bilateral disbursements to RMNCH and to PNH

Disbursements to recipients other than named countries took two forms: disbursements to a named region (e.g. ‘South America, regional’) or to ‘Bilateral, unspecified’. For RMNCH estimates, such disbursements were distributed between individual recipient countries based on their year-specific share of RMNCH disbursements to recipient countries in the region^4^, or, in the case of ‘Bilateral, unspecified’ disbursements, their share of bilateral disbursements to all countries^1^.

Regional and unspecified bilateral disbursements for PNH were assigned to recipient countries in proportion to their receipt of country-specific funding for PNH over the entire 2003–13 period. Unlike the approach for RMNCH funding, we did not perform the operation separately for each year because of the dearth of records mentioning PNH, especially in the early years.

#### Worked examples of RMNCH estimates

**Figure 2:**
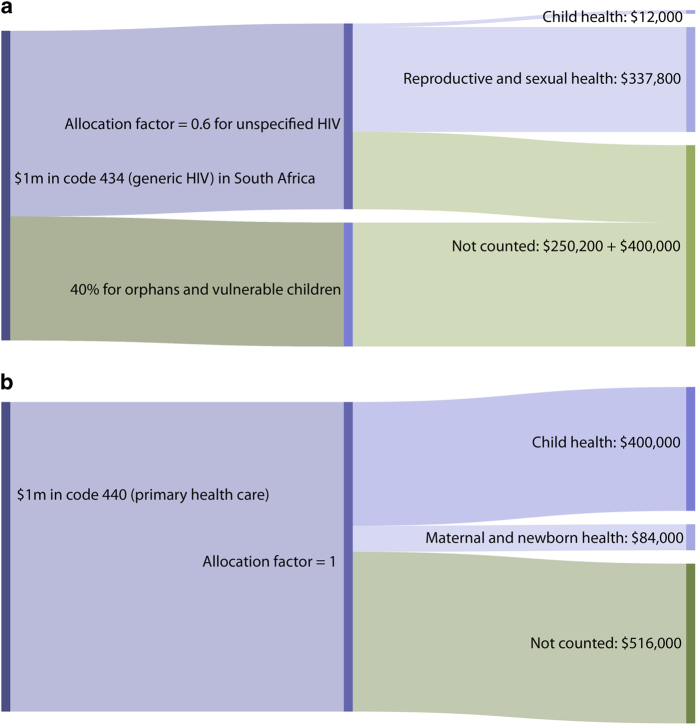
Examples of how disbursement totals are calculated.

Figure 2a,b provide examples of hypothetical disbursement calculations. A project for $1m for generic HIV/AIDS programming in South Africa that was 60% for unspecified HIV care and 40% for orphans (excluded in the coding scheme), would receive RMNCH code 434 and an allocation factor of 0.6. The proportions of the HIV-positive population in South Africa that are aged under five, or are women aged 15+, are estimated at 2.0 and 56.3% respectively. Our calculations thus result in an estimate that this record provided $12,000 to child health, $337,800 to reproductive and sexual health, and no funding to maternal and newborn health. The 40% of the disbursement to orphans ($400,000) and the remaining part of the general population disbursement ($250,200) are not counted in our estimates of ODA+ for RMNCH (Fig. 2a).

For a disbursement record of $1m for primary health care, receiving RMNCH code 440 and allocation factor 1, $400,000 is considered to support child health, $84,000 to support maternal and newborn health, and no funding is considered to support reproductive and sexual health. The remaining $516,000 is not counted as supporting RMNCH (Fig. 2b).

#### Code availability

The generation of the fully coded Countdown ODA+ Dataset (Data Citation 1) cannot be replicated, as it involved human agents applying a set of descriptive rules to assign codes. Although this framework was consistently applied (see Technical Validation below), it was not deterministic.

We make available the SQL Server Management Studio code we used for the data matching, including the data cleaning (Supplementary File 4).

## Data Records

The Countdown ODA+ Dataset is deposited at http://datacompass.lshtm.ac.uk/320/asazipped.txtfile. Table 7 summarises the sources of the data in the Dataset. Manipulations are described in the sections ‘Coding records for RMNCH and for PNH’, ‘Matching the RMNCH-coded records to 2015 full CRS, and coding unmatched records in the 2015 full CRS’, ‘Reviewing the final dataset with regard to known inconsistencies’ and ‘Technical validation’.

There is a smaller version of the dataset with expenditure values for child health, maternal and newborn health and reproductive and sexual health aggregated by year, donor, recipient, CRS purpose category and disbursement channel, also available at http://datacompass.lshtm.ac.uk/320/ in .csv, .sas7bdat, .sav, .dta and .xlsx formats.

The data deposit also contains a user guide, and a data dictionary that describes the fields in the dataset.

There is also an interactive data deposit based on the same expenditure data for child health, maternal and newborn health, and reproductive and sexual health, through the Tableau Public interface at https://public.tableau.com/profile/ardito#!/vizhome/WorldRMNCH/DisbursementsRMNCH.

## Technical Validation

It is not possible to validate the codes assigned against a ‘gold standard’. However, we took several measures aimed at ensuring accuracy and consistency. Round 1 had two coders for MNCH who coded the same projects blinded to each other, then compared results and resolved inconsistencies. We assessed the agreement between the two coders and found that less than 3% of the total variance was due to difference between the two coders^7^.

In round 5, two coders each coded for PNH the records identified by the key-term search. Agreement was very high both for whether records exclusively benefitted newborns, also benefitted other population groups, or were misclassified (98.4%), and for whether correctly classified projects supported research or non-research programmatic and advocacy activities (97.3%). Discrepant codes were reconciled through discussion^6^.

In round 6 four coders assigned codes for RMNCH. We assessed reliability between coders using Krippendorf’s alpha on a sample of 1,270 records. This gave a score of over 0.9 among three of the coders (equivalent to ‘almost perfect’ agreement on Landis and Koch’s arbitrary scale of agreement for categorical data)^16^, and a score under 0.7 for the fourth. All records coded by the fourth coder were re-coded by one of the other coders, and differences resolved by discussion^1^.

After coding in round 7 we conducted several checks of consistency in coding over time resulting from potential differences in the application of codes by different coders. We investigated the frequency of records under various purpose codes that were given non-zero RMNCH codes; and the frequency of RMNCH codes assigned across the years. Frequencies were graphed and investigated visually. For example, in this way we discovered that in 2008 and 2009 there had been far fewer records with purpose code 12263 (tuberculosis control) assigned non-zero RMNCH codes than in other years. We investigated the corresponding records to check whether the RMNCH codes had been correctly assigned, and made corrections to 662 records. In another example, we knew that vaccination for yellow fever had in some years been coded as entirely benefitting children (RMNCH code 415); such records were identified and assigned the correct code for vaccinations benefitting the general population (436).

Across all the investigations we made in round 7, this editing process changed the RMNCH codes assigned to 9,694 records (0.5%), amounting to a net increase of 4.2% of total RMNCH disbursement value (USD 3,977 m).

### Limitations

Our coding depends on how donors describe their projects, which could potentially give rise to systematic bias in classifications by donor. Donor descriptions of similar projects might change over time to emphasise issues prominent in the discourse at the time of reporting. For example, there was a shift from the term ‘MCH’ for maternal and child health, to the term ‘MNCH’, but we cannot say whether this made any difference to the content of programmes. The degree of detail, descriptiveness and length of descriptions varies greatly between donors and is a systematic difference. While more detailed descriptions can increase coding precision, they might also increase coder fatigue and increase the risk of mistakes; we attempted to mitigate this risk through the use of flags for RMNCH coding as described above.

The CRS only includes funds reported to the OECD, and does not include ODA from countries that do not report to the CRS such as China and Brazil, or funds from non-governmental organisations and private foundations that choose not to report to CRS, which can be substantial. Data on some of these funds are available from the AidData database. Domestic funds for RMNCH are also not included.

There is potential for reasonable disagreement over our RMNCH codes and disbursements rules. They are conceptually coherent but exclude some arguably relevant activities (such as water and sanitation), and include others whose inclusion is debatable (e.g. breast cancer within the sexual health code). We have tried to be clear as to what goes in each category, but ultimately we recognise that other frameworks with different inclusion/exclusion/allocation rules could be equally valid for estimating disbursements to RMNCH. While we believe that the large majority of projects were reliably coded, there will always be records for which the RMNCH code or allocation factor are not clear cut, and which will therefore be more prone to inconsistent coding.

## Usage Notes

Reuse potential includes:

- In-depth investigations of donor-recipient relationships over time.
- Recipient- or donor-specific investigations of funding for particular health areas.
- Analysis of funding to specific reproductive, maternal or child health activities (e.g. immunisation or HIV funding) by donor and by recipient country over time.
- Comparison of funding to RMNCH or any component therein to funding to other health areas or other sectors.

The data can be linked to other data relating to characteristics of recipient countries (e.g. health outcomes, socio-political context, levels of domestic funding) to enable further econometric analysis of determinants of aid, and effects of aid.

Frequent changes made to the records reported to the CRS and the absence of unique identifiers for records within the CRS mean it is unlikely that users will be able to reliably match the present records to future iterations of the CRS. However, we provide the code for conducting this matching in case this proves possible.

## Additional Information

**How to cite this article**: Grollman, C. *et al.* Developing a dataset to track aid for reproductive, maternal, newborn and child health, 2003–2013. *Sci. Data* 4:170038 doi: 10.1038/sdata.2017.38 (2017).

**Publisher**’**s note**: Springer Nature remains neutral with regard to jurisdictional claims in published maps and institutional affiliations.

## Supplementary Material



Supplementary File 1

Supplementary File 2

Supplementary File 3

Supplementary File 4

## Figures and Tables

**Table 1:** RMNCH codes and disbursement rules for CH, MNH and R*^7^.

RMNCH code	Nature of the project	CH fraction	MNH fraction	R* fraction
0	Exclude			
100	General budget support	Country value	Country value	Country value
200	Health sector budget support	20%	12%	
300	Health basket-funding	40%	8%	
411	Child malaria	100%		
412	HIV/AIDS—prevention of mother-to-child transmission	100%		
413	Nutrition in children	100%		
414	Integrated management of childhood illnesses	100%		
415	Immunisation—excluding polio	100%		
416	Immunisation—polio	100%		
417	Non-specified infectious diseases in children	100%		
418	Non-specified child health	100%		
419	Childhood HIV/AIDS	100%		
421	Reproductive, maternal and neonatal health		100%	
422	Family planning			100%
423	Sexual health			100%
424	Sexual/reproductive health—mixed		50%	50%
431	Maternal and child health	50%	50%	
432	Malaria—generic	Region value	15%	
433	Maternal and child malaria	55%	10%	
434	HIV/AIDS—generic	Country value		Country value
435	Nutrition—generic	Country value		
436	Non-specified infectious diseases	50%	50%	
437	Sexually transmitted infections—generic			Region value
440	General health system—primary health care	40%	8.4%	
450	General health system—hospital level care	11.3%	13.3%	
460	General health system—policy/all levels	19.8%	11.8%	
This table shows the proportion of the disbursement value for each RMNCH code that is counted as supporting child health, maternal and newborn health and reproductive and sexual health. Country/regional values were calculated based on sources described in the section ‘Proportions of disbursements assigned to R*, MNH, CH’.				
CH, child health; MNH, maternal and newborn health; RMNCH, reproductive, maternal, newborn and child health; R*, reproductive and sexual health.				

**Table 2:** Descriptions of RMNCH codes.

**RMNCH code**	**Summary**	**Description**
0	Exclude	Not related to RMNCH according to our definition. This typically includes activities related to: research, dentistry, tobacco and alcohol/drugs, and non-health-related activities. Research activities are also coded 0, as are conferences unless clearly oriented toward policy or implementation rather than science (e.g. Women Deliver). Core contributions from donors to multilateral agencies such as UNAIDS are coded 0. Everything reported as debt relief (purpose code 600XX) or donor-country promotional activities (purpose code 99820) receive a 0 code.
100	General budget support	Funds disbursed to the central bank or regional governments of the recipient country with no earmarking. Funds should be associated with poverty reduction as opposed to balance of payment support. Includes poverty reduction support credits (PRSCs) but not structural adjustment loans or macroeconomic stabilisation grants. Includes any generic/unspecified ‘budget support’ records.
200	Health sector budget support	Funds disbursed to the central bank or regional governments of the recipient country but earmarked specifically for the health sector. Funds are not earmarked to areas within the health sector. Includes ‘block grants’ to the health sector.
300	Health basket-funding	Funds earmarked to specific areas within the health sector e.g. primary health care. Also includes basic package of health services.
411	Child malaria	Entire project is oriented to malaria activities targeting children from one week up to five years.
412	HIV/AIDS—prevention of parent-to-child transmission	Entire project is oriented to the prevention of parent-to-child transmission of HIV.
413	Childhood nutrition	Entire project is oriented to nutrition activities targeting children under five. Includes breastfeeding and supplementary feeding. Excludes food security, food aid, and school feeding. Unspecified ‘nutrition’ projects are coded as 435 (Nutrition—generic).
414	Integrated management of childhood illnesses	Entire project is oriented to integrated management of childhood illnesses (IMCI). Includes integrated community case management (ICCM), integrated management of newborn and child illness (IMNCI), community integrated management of childhood illnesses (CIMCI), ‘EPI [expanded programme of immunisation] +malaria’, young child survival and development (YCSD).
415	Immunisation—excluding polio	Entire project is oriented to immunisation activities and does not specify that the project will only include polio immunization. Includes generic/unspecified vaccine/immunisation projects. Includes tuberculosis unless specified for adults. Excludes vaccines in emergency situations (e.g. cholera, yellow fever) or other vaccines that benefit the general population (436). Excludes human papillomavirus (HPV) vaccination (423). Includes ‘cold chain’ etc in 120 codes unless specified for blood (450).
416	Immunisation—polio	Entire project is oriented to polio immunisation activities.
417	Non-specified infectious diseases in children	Entire project is oriented to infectious disease activities other than malaria or HIV/AIDS targeting children under five. The activities target either a single infectious disease or multiple infectious diseases.
418	Non-specified child health	Entire project is oriented to health activities targeting children under five. The activities do not fall within any of the previous child health categories or are not specified. Includes activities to support children’s hospitals and paediatric wards; unspecified child protection listed in 120/130; child survival; and unspecified ‘child health’ projects and health projects for ‘children’.
419	Childhood HIV/AIDS	Entire project is oriented to treatment of paediatric HIV/AIDS.
421	Reproductive, maternal and neonatal health	Entire project is oriented to maternal and/or neonatal health activities including safe motherhood, care during childbirth and newborn care. Includes training of midwives and obstetricians, congenital syphilis and fistula. Excludes family planning 422, sexual health and sexual and gender-based violence 423, activities related to sexually transmitted infections 437, and all projects referring to ‘reproductive commodities’ (e.g. not contraceptives) unless further information is provided to indicate that the commodities fall within our definition of maternal and newborn health, such as clean delivery kits and other commodities for safe motherhood (else 424). Excludes projects which also mention children, which should be coded as 431. Includes unspecified 13020 projects, but vague ‘reproductive health’ projects are coded as 424 (sexual/reproductive health—mixed).
422	Family planning	Project is oriented to family planning including the provision of and counselling in contraceptive commodities, abortion services, infertility drugs and procedures, and information, education and communication (IEC) activities that support or promote family planning.
423	Sexual health	Entire project is focused on sexual health. Includes behaviour change programmes for safer sexual behaviour, cancers of the reproductive system (including breast, cervical, ovarian, uterine) and HPV vaccination, and sexual and gender-based violence (e.g. rape, incest, sexual trafficking and sexual exploitation) if listed under health or reproductive health purpose codes (120/130) or contains a clear description as addressing a health and not a human rights or other social service aspect if included in the government and civil society codes (150) or other social services codes (160). Includes projects related to female genital mutilation listed in 120/130.
424	Sexual/reproductive health—mixed	Includes adolescent/youth sexual and reproductive health programmes or any other programmes that specify SRHR (sexual and reproductive health and rights). Generally includes projects with CRS purpose code 13030 implemented by International Planned Parenthood Federation (IPPF) and many from UNFPA. Use for ‘mixed’ interventions that straddle across code 421 and other RMNH codes—but not code 431. Includes vague ‘reproductive health’ projects and unspecified projects with purpose code 13081; includes vague projects with purpose code 13040 where donor is UNICEF.
431	Maternal and child health	Entire project is oriented to maternal and child health (children older than one week). Includes neonatal only if child is also included. Excludes specific malaria 433 and nutrition 435 projects.
432	Malaria—generic	Entire project is oriented towards malaria without specifying the target population.
433	Maternal/child malaria	Entire project is focused on malaria prevention or treatment activities for women during pregnancy, childbirth and the postnatal period (1 week) for newborns, and projects for both mothers and children under five. Includes all ITN projects, which are assumed to be for mothers and children only. Programmes for children under five only are coded 411.
434	HIV/AIDS—generic	Entire project is oriented towards HIV/AIDS without specifying the target population. TB integrated with HIV is included. Includes abstinence etc if described as for HIV prevention. Include generic ‘high-risk populations’ or similar; include sex workers, using an allocation factor if named alongside other high-risk groups; exclude if specifically for men having sex with men or injecting drug users. To the extent possible, exclude projects targeting the socio-economic causes and consequences of HIV, such as livelihoods, education, and orphans and vulnerable children (OVC).
435	Nutrition	Entire project is focused on nutrition activities for women and children. If not explicitly mentioned, both general ‘nutrition’ projects and targeted food supplementation to malnourished persons are assumed to target women and children only. Excludes food security, food aid and school feeding, and excludes projects that mention these alongside ‘nutrition’. Agriculture projects in purpose code 311 is that they are excluded.
436	Non-specified infectious diseases and other health conditions	Project is focused on health activities for which children are likely to be a beneficiary with the same probability as the rest of the population wherein the project does not specify the target group. Includes combined projects targeting multiple infectious diseases (e.g. projects targeting Malaria, TB and HIV jointly). Also includes general population vaccines including emergencies (e.g. cholera), avian flu, land mine clearing and awareness but not solely advocacy or economic support to victims, arsenic poisoning prevention. Includes water, sanitation and hygiene (WASH) projects in 140 that mention a targeted disease (e.g. cholera, diarrhoea). Includes eye health projects (in 120) except those addressing non-child problems such as cataracts (0). Excludes diabetes, alcoholism, kidney disease and non-communicable diseases generally unless likely to affect children under five.
437	Sexually transmitted infections—generic	Entire project is focused on sexually transmitted infections (STIs) without specifying the condition or the target population. Includes testing, prevention, treatment and care of STIs, reproductive tract infections and other gynaecological morbidities. Includes HIV alongside STIs, but not HIV alone.
440	General health system—primary health care	Project supports the delivery of integrated services at the primary or basic level of the health system. Such projects do not target a specific disease or area of health. Includes training, infrastructure or resourcing (e.g. drugs) explicitly for primary health care. Includes WASH activities whose primary stated purpose is to improve human health—not WASH infrastructure alone—as shown by inclusion in the health purpose codes (120); if included in the water purpose codes (140XX) or under the humanitarian purpose codes (720XX), a WASH project would get 440/450/460 if providing WASH for health facilities, 436 if addressing a named disease, and otherwise 0. WASH for schools is excluded; a project for ‘schools and communities’ would get 440 with AF 0.5. Includes humanitarian activities defined vaguely as ‘health’ or implemented by a humanitarian organization whose primary area of activity is health and community health. Includes ‘health education’ projects targeting the population.
450	General health system—hospital level care	Project supports the delivery of integrated services at the hospital or secondary level of the health system. Such projects do not target a specific disease or area of health. Includes blood transfusion services. Includes training, infrastructure or resourcing (e.g. drugs) explicitly for hospital-level care.
460	General health system—policy/all levels	Project supports health system development rather than a specific disease, or demographic group. This could be either at the administrative level (i.e. policy, planning and monitoring) or at the level of service delivery (i.e. health providers). Such projects typically provide financial support for health infrastructure, human resources (which includes salaries as well as training of nurses and doctors including higher education for medical school), drug provision and management, medical equipment, policy development and monitoring systems (e.g. information systems), except where these are more specifically described as coming under 440/450. Includes ‘health sector reform’ projects, core support to international health service delivery NGOs, advocacy and health promotion. Excludes funding for policy in more specific codes, which should take the more specific code. Includes all health insurance projects. Includes ‘health education’ where the object is providers of such education.
999	Disbursement blank/zero	Disbursement is zero or not reported.
AF, allocation factor; RMNCH, reproductive, maternal, newborn and child health.		

**Table 3:** Descriptions of the fields in the Creditor Reporting System (CRS) reports that were consistently used for RMNCH coding.

CRS field name	Function
Donor name	The source of the disbursement, a bilateral agency, multilateral agency, global health initiative or private foundation.
Project title	This is the title of the project.
Short description	This provides a short description of the project.
Long description	This provides a longer description of the project.
Purpose code	This is a code, from the standard reporting template of the CRS, assigned by the donor to indicate the area of expenditure. There are 203 purpose codes, grouped into 26 main sectors, two of which relate directly to health: 120 (Health) and 130 (Population Policies/Programmes and Reproductive Health). The full list of purpose codes is available from the OECD (http://www.oecd.org/dac/stats/dacandcrscodelists.htm, accessed on 1 April 2016).

**Table 4:** CRS purpose codes and assumed RMNCH codes in the absence of any further descriptive information.

**Purpose code**	**Assumed RMNCH code in absence of further information**
12110—Health policy and administrative management	460—General health system—policy/all levels
12181—Medical education/training	460
12182—Medical research	0—Exclude
12191—Medical services	460
12220—Basic health care	440—General health system—primary health care
12230—Basic health infrastructure	440
12240—Basic nutrition	435—Nutrition
12250—Infectious disease control	436—Non-specified infectious diseases
12261—Health education	460
12262—Malaria control	432—Malaria—generic
12263—Tuberculosis control	436
12281—Health personnel development	460
13010—Population policy and administrative management	0
13020—Reproductive health care	424—Sexual/reproductive health—mixed
13030—Family planning	422—Family planning
13040—Sexually transmitted disease control including HIV/AIDS	437—Sexually transmitted infections—generic
13081—Personnel development for population and reproductive health	424
CRS, Creditor Reporting System; RMNCH, reproductive, maternal, newborn and child health.	

**Table 5:** Iterations of coding conducted across 7 rounds over the course of the Countdown initiative.

**Coding round**	**Areas of health**	**Sources and years of newly coded data (number of records)**	**Years of data updated**	**Number of coders**	**Reference**
1	MNCH	CRS 2003–04GAVI 2003–04GFATM 2003–04 (>80,000)	–	2 double coding	Powell-Jackson *et al.*^7^
2	MNCH	CRS 2005–06GAVI 2005–06GFATM 2005–06 (96,946)	–	1	Greco *et al.*^2^
3	MNCH	CRS 2007–08GAVI 2003–07GFATM 2003–04 (385,771)	2003–07 for IDA2006 Italy2004–05 Finland2005–06 UNFPA2003–04 and 2006 UNAIDS	1	Pitt *et al.*^5^
4	RMNCH	CRS 2009–10 (470,310)	–	1	Hsu *et al.*^4^; Hsu *et al.*^3^
5	PNH	CRS 2002–10 (4,584†)	–	2 double coding	Pitt *et al.*^6^
6	RMNCH (2009–12)MNCH (2003–12)	CRS 2011–12 (507,954)	–	4 coding separate sections, with consistency checks	Arregoces *et al.*^1^
7	RMNCH	CRS 2013 (231,398)GAVI 2003–06 (1,190)	2003–122003–08 for R*	1	Grollman *et al.* 2016
	PNH	CRS 2003–13 (15,062†)	–	2	Pitt *et al.*^11^ (forthcoming)
CRS, Creditor Reporting System; GAVI, the Vaccine Alliance; GFATM, Global Fund to Fight AIDS, IDA, International Development Association; PNH, prenatal and neonatal health; RMNCH, reproductive, maternal, newborn and child health; R*, reproductive and sexual health; Tuberculosis and Malaria; MNCH, maternal, newborn and child health; †, identified by key term searching prior to coding.					

**Table 6:** Sources of RMNCH codes in the final dataset.

**Source of RMNCH code**	**Number of records**		**RMNCH value (2013 US$ m)**	
**Zero/null disbursement**	**3,92,904**	**18.5%**	**0**	**0.0%**
**2015 full CRS record matched to records coded in rounds 1–7**	**12,05,434**	**56.8%**	**88,256.3**	**90.2%**
*code reviewed for known inconsistencies*	9645	0.5%	3,911.7	4.0%
**2015 full CRS record did not match any record coded in rounds 1–7 and coded in preparation of this dataset**	**5,24,185**	**24.7%**	**8,719.6**	**8.9%**
*code reviewed for known inconsistencies*	49	0.0%	65.7	0.0%
**2015 full CRS sub-total**	**21,22,523**	**99.9%**	**96,975.8**	**99.1%**
**GAVI 2003–06 coded in preparation of this dataset**	**1,190**	**0.1%**	**884**	**0.9%**
**Total**	**21,23,713**		**97,859.8**	
Table shows numbers of records in the Countdown ODA+ Dataset that were coded for RMNCH in rounds 1–7 of the Countdown initiative, were coded in preparation of the final dataset, had zero or null disbursement value, and came from GAVI for the years 2003–06.				
CRS, Creditor Reporting System; GAVI, the Vaccine Alliance; RMNCH, reproductive, maternal, newborn and child health.				

**Table 7:** Input data sources brought together in preparing the present dataset.

**#**	**Source**	**Number of records**	**Dates covered**	**Use**	**Processing**
1	Coded records from 7 rounds of coding in the Countdown initiative	1,438,307	2003–2013	Provided RMNCH codes to match to (2)	As described in sections on matching, coding and calculating disbursements
2	2015 full CRS, from OECD Creditor Reporting System	2,122,523	2003–2013	Data on ODA+ disbursements	
3	Records from the Vaccine Alliance (GAVI)	1,190	2003–2006		
4	Reference data on populations from multiple sources (see Table 4)	–	2003–2013	Informed calculation of disbursement values for RMNCH following combination of (1) with (2) and (3) and further coding	
CRS, Creditor Reporting System; ODA+, official development assistance and private grants; OECD, Organisation for Economic Cooperation and Development; RMNCH, reproductive, maternal, newborn and child health.					
